# Polymorphisms of Fat Mass and Obesity-Associated Gene in the Pathogenesis of Child and Adolescent Metabolic Syndrome

**DOI:** 10.3390/nu15122643

**Published:** 2023-06-06

**Authors:** Yongyan Song, Henry Wade, Bingrui Zhang, Wenhao Xu, Rongxue Wu, Shujin Li, Qiaozhu Su

**Affiliations:** 1 Central Laboratory, Clinical Medical College & Affiliated Hospital of Chengdu University, Chengdu 610106, China; songyongyan@cdu.edu.cn; 2 Institute for Global Food Security, School of Biological Sciences, Queen’s University Belfast, Belfast BT9 5DL, UK; 3 Clinical Medical College, Chengdu University, Chengdu 610106, China; 4 Section of Cardiology, Department of Medicine, Biological Sciences Division, University of Chicago, Chicago, IL 60637, USA

**Keywords:** metabolic syndrome, fat mass and obesity-associated gene, genetic polymorphism, child, adolescent, rs9939609 polymorphism, rs9930506 polymorphism

## Abstract

Childhood metabolic syndrome (MetS) is prevalent around the world and is associated with a high likelihood of suffering from severe diseases such as cardiovascular disease later in adulthood. MetS is associated with genetic susceptibility that involves gene polymorphisms. The fat mass and obesity-associated gene (*FTO*) encodes an RNA N6-methyladenosine demethylase that regulates RNA stability and molecular functions. Human *FTO* contains genetic variants that significantly contribute to the early onset of MetS in children and adolescents. Emerging evidence has also uncovered that *FTO* polymorphisms in intron 1, such as rs9939609 and rs9930506 polymorphisms, are significantly associated with the development of MetS in children and adolescents. Mechanistic studies reported that *FTO* polymorphisms lead to aberrant expressions of *FTO* and the adjacent genes that promote adipogenesis and appetite and reduce steatolysis, satiety, and energy expenditure in the carriers. The present review highlights the recent observations on the key *FTO* polymorphisms that are associated with child and adolescent MetS with an exploration of the molecular mechanisms underlying the development of increased waist circumference, hypertension, and hyperlipidemia in child and adolescent MetS.

## 1. Background

### 1.1. Incidence of Pediatric Metabolic Syndrome

Cardiovascular and cerebrovascular diseases are currently the world’s leading cause of death [1,2,3]. There are multiple risk factors that contribute to the pathogenesis of cardiovascular and cerebrovascular diseases, including aging, male gender, smoking, hyperuricemia, hyperhomocysteinemia, obesity, diabetes, and metabolic syndrome, i.e., hyperlipidemia and hypertension. According to the definitions of the National Cholesterol Education Program’s Adult Treatment Panel III (NCEP ATP III) and the International Diabetes Federation (IDF), MetS consists of five essential components: abdominal obesity, hypertension, hyperglycemia, hypertriglyceridemia, and hypo-high-density lipoproteinemia, which are all risk factors for cardiovascular and cerebrovascular diseases. There are currently no separate diagnosis criteria for pediatric MetS, and childhood MetS has the same criteria set as that of adults defined by NCEP ATP III and IDF with minor modifications [4,5,6,7,8,9]. Both sets of criteria employ waist circumference (WC), systolic blood pressure (SBP), diastolic blood pressure (DBP), plasma triglycerides (TGs), high-density lipoprotein cholesterol (HDL-C), and fasting blood glucose (FBG) as diagnostic indexes (Table 1). The prevalence of MetS in children and adolescents ranges from 6–12% in developed countries in Europe and North America [4,5,6] and 2–6% in developing nations such as China and India [7,8,9]. In a study of European children with a median age of 11.2 years and from eight European countries (Germany, Italy, Spain, Belgium, Sweden, Estonia, Hungary, and Cyprus), the researchers recently reported that about 6% of the children had MetS [4]. In the United States, DeBoer and colleagues [5] determined the prevalence of MetS among 4600 adolescents aged 12–19 years from different regions in 2019 and found that the overall prevalence was 6.25%, 6.31%, 7.57%, and 11.42% in northeast, west, south and midwest regions, respectively. Zhu et al. [7] estimated the MetS status among 15,045 children and adolescents aged 7–18 years across seven Chinese provinces in 2020 and reported the overall MetS prevalence was 2.3%. Ten years ago, Andrabi et al. [8] evaluated that the prevalence of MetS in a cohort of Indian children and adolescents aged 8–18 years and observed that the prevalence of MetS was 3.8% in girls and 3.9% in boys. During childhood and adolescence, MetS and its components are related to an increased incidence of metabolic diseases such as diabetes, cardiovascular disease, cancer, and adulthood infertility [10,11,12]. As a complex disease with multiple components, pediatric MetS is associated with various risk factors including genetic variation, dietary habits, physical activity, and air pollution [13,14]. In recent years, more and more susceptibility genes and genetic variants linked to childhood MetS were identified by scientists around the world.

### 1.2. Fat Mass and Obesity-Associated Gene in Pediatric MetS

The fat mass and obesity-associated gene (*FTO*) encodes an N6-methyladenosine (m6A) demethylase and belongs to the superfamily of Fe (II)- and 2-oxoglutarate-dependnet dioxygenases. Human *FTO* is localized in chromosome 16q12.2 and has a length of 420 kb containing eight introns and nine exons; alternative posttranscriptional splicing of human *FTO* mRNA yields twelve protein-coding transcripts. The main function of *FTO* is to demethylate m6A of different RNA species, such as mRNAs and snRNAs, thereby regulating the stability and molecular functions of such RNAs. *FTO* has a strong association with obesity in humans; *FTO* was first identified to be an obesity sensitivity gene according to a genome-wide association study in 2007 [18] and *FTO* was shown to be highly expressed in the abdominal and subcutaneous adipose tissues of obese individuals [19,20]. Multiple single nucleotide polymorphisms (SNPs) in this gene were subsequently identified to be strongly correlated with obesity indexes, including body mass index (BMI) and WC. As a result, it was named as the “fat mass and obesity-associated gene” and received increasing and extensive attention in recent years.

The relation between *FTO* and adiposity has been further strengthened by data demonstrating *FTO* as a regulator of adipogenesis and adipocyte differentiation. Overexpression of *FTO* induced obesity in mice, associated with increased triglyceride deposition, whilst knockdown of *FTO* caused adipose tissue atrophy [21,22,23]; furthermore, *FTO* overexpression promoted adipogenesis in preadipocytes, murine embryonic fibroblasts, and porcine intramuscular preadipocytes [23,24,25,26]. This effect occurred because *FTO* inhibition reduced the expression of Janus kinase 2 and activated signal transducer and activator of transcription 3 by phosphorylation [23,24,25,26]. These effects contributed to impaired transcription of CCAAT enhancer binding protein β (C/EBPβ), an essential gene for early adipocyte differentiation. Furthermore, reduced expression of *FTO* impaired adipogenesis during mitotic clonal expansion, an essential prerequisite for adipocyte differentiation, by increased expression of runt-related transcription factor 1 (RUNX1) [25]; it was also suggested that *FTO* inhibition impaired cell cycle progression at the S to G_2_ phase by reduction of cyclin A2 as well as cyclin-dependent kinase 2 (CDK2) [23,27]. This review highlights the recent advances in studies that investigate the relationships between *FTO* variants and MetS in childhood and adolescence as well as the underlying mechanisms. The experimental findings discussed in this review fully demonstrate a close relationship of *FTO* polymorphisms with obesity and the relevant metabolic disorders.

## 2. Some Genetic Polymorphisms of *FTO* Increase Susceptibility to MetS

**Figure 1:**
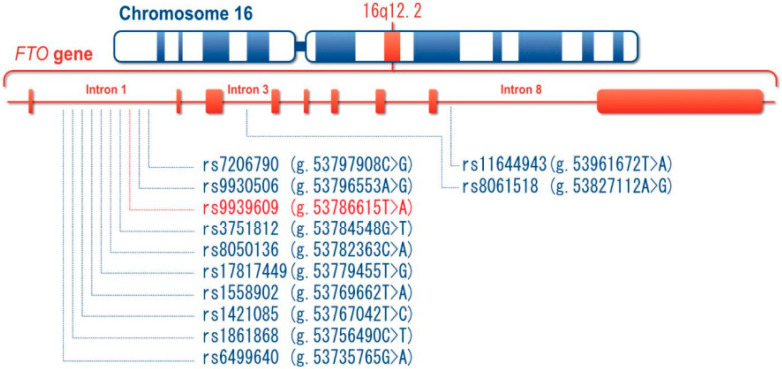
The genetic landscape of the *FTO* variants associated with MetS and/or its components in children and adolescents. *FTO*, fat mass and obesity-associated gene; MetS, metabolic syndrome.

*FTO* is highly polymorphic; many *FTO* polymorphic loci can be found in the dbSNP database established by the National Center for Biotechnology Information (NCBI). A lot of research indicates that some *FTO* variants, mostly those loci within intron 1 and rarely in other introns, are associated with MetS and/or its components in children and adolescents. Based on the locations in *FTO*, these genetic loci are categorized into promoter polymorphisms (e.g., rs62048369, rs779839225, and rs367716710), exonic polymorphisms (e.g., rs79206939, rs139577103, and rs140101381), and intronic polymorphisms (e.g., rs9939609, rs17817449, and rs1421085). Some *FTO* polymorphic sites, especially those localized within the first intron, have been shown to be significantly related to MetS and/or its biomarkers, i.e., WC, SBP/DBP, TG, HDL-C, and FBG, in children and adolescents (Figure 1). Several of the most frequent polymorphisms are discussed below.

### 2.1. The SNPs of FTO in MetS: rs9939609 and rs9930506 Polymorphisms

#### 2.1.1. The rs9939609 Polymorphism

Among the polymorphisms of *FTO*, the rs9939609 polymorphism was the most extensively investigated locus displaying a significant relationship with MetS and/or its components. The rs9939609 (g.53786615T>A) variant is localized within intron 1 of *FTO*, with thymine (T) as the major allele and adenine (A) as the minor allele. Based on the records of SNP databases such as NCBI’s dbSNP, Ensembl, and VannoPortal, the frequencies of the minor allele A range from 0.39 to 0.44 among European and American Caucasians, 0.11 to 0.20 among East Asians, 0.48 to 0.49 among Africans, and 0.25 to 0.26 among Latin Americans. In 2007, Frayling et al. reported that human subjects with the AA or AT genotype of the rs9939609 genetic polymorphism had 1.67 times higher odds of adiposity and 3 kg heavier body weight when compared to the TT homozygotes in 13 European cohorts with 38,759 participants aged 28–74 years [18]. Almén et al. [28] investigated the genome-wide DNA methylation profile among female preadolescents with different variations of the rs9939609 genetic polymorphism and identified 20 differentially methylated obesity-related loci.

**Figure 2:**
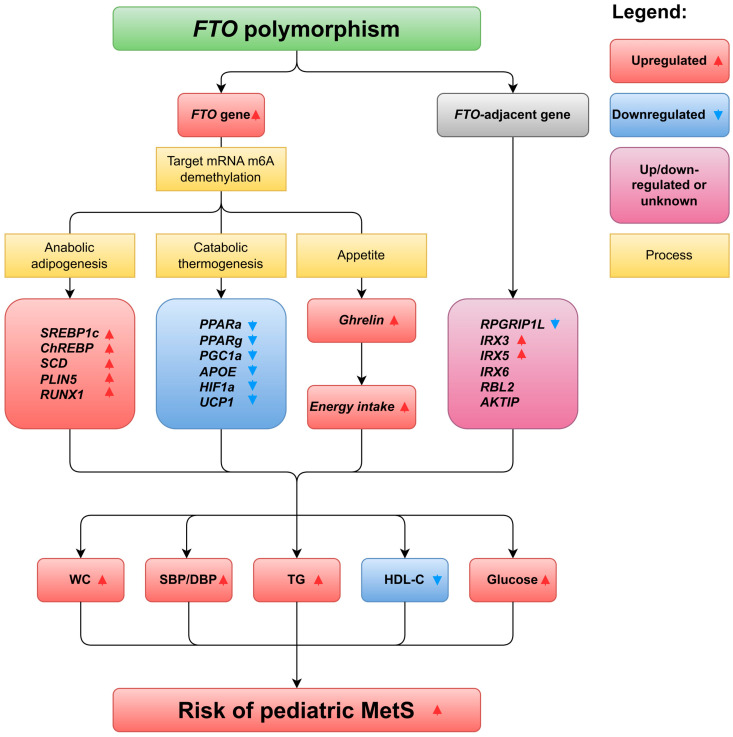
The pathophysiological roles of *FTO* polymorphisms in childhood MetS. *FTO*, fat mass and obesity-associated gene; MetS, metabolic syndrome; WC, waist circumference; SBP, systolic blood pressure; DBP, diastolic blood pressure; TG; triglyceride; HDL-C, high-density lipoprotein cholesterol; FBG, fasting blood glucose; SREBP1c, sterol regulatory element-binding protein 1c; ChREBP, carbohydrate responsive element-binding protein; SCD, stearoyl-CoA desaturase; PLIN5, perilipin 5; RUNX1, runt-related transcription factor 1; PPARα, peroxisome proliferator-activated receptor alpha; PPARγ, peroxisome proliferator-activated receptor gamma, APOE, apolipoprotein E, HIF1α, hypoxia-inducible factor 1 alpha; UCP1, uncoupling protein 1, *RPGRIP1L*, retinitis pigmentosa GTPase regulator-interacting protein-1-like; *IRX3/5/6*, Iroquois homeobox protein 3/5/6; *RBL2*, retinoblastoma-like 2; *AKTIP*, AKT-interacting protein.

The mechanism by which SNP rs9939609 increases risk of pediatric MetS has not been fully elucidated. The first potential mechanism by which *FTO* polymorphisms affect MetS susceptibility could be that *FTO* variants cause aberrant expression of the *FTO* gene, which in turn disturbs the methylation status of *FTO*-targeted mRNAs and other non-coding RNAs, resulting in metabolic disorders as well as MetS (Figure 2). Indeed, Skuladottir et al. observed that the subjects with the AA genotype of the rs9939609 polymorphism had greater blood levels of stearoyl-CoA desaturase (SCD) mRNAs and enzymatic activities, a key enzyme in lipogenic pathway, than those with the TT genotype in a population of young healthy subjects [29]. Villalobos-Comparán and colleagues detected *FTO* transcription in 31 biopsies of subcutaneous fat tissue of Mexican women, and the results showed that the A allele carriers of the rs9939609 variant expressed more *FTO* mRNA than those with the TT genotype (2.41 vs. 1.46, *p* = 0.047) [20,30]. Berulava et al. assessed the *FTO* expression pattern by using skin biopsies and blood samples collected from 18 normal-weight subjects and observed that the A allele carriers of the rs9939609 polymorphism had 1.38 (95% CI 1.31–1.44) and 1.31 (95% CI 1.23–1.39) times more abundant *FTO* transcripts than those with the TT genotype in blood samples and skin biopsies, respectively [31]. Karra et al. analyzed the *FTO* expression in peripheral blood cells collected from subjects with different rs9939609 genotypes, and the data indicated that the subjects with the AA genotype exhibited increased *FTO* mRNA [32]. However, another two studies did not detect any significant difference between the rs9939609 polymorphism and *FTO* mRNA expression in human subcutaneous adipose and skeletal muscle biopsies, respectively [19,33]. To date, the relation between *FTO* polymorphisms and *FTO* expression is still elusive, and more research is required to clarify the issue. These observations may indicate that increased transcripts of *FTO* in A allele carriers of the rs9939609 polymorphism contribute to a higher risk of MetS in this population.

#### 2.1.2. The rs9930506 Polymorphism

Another intronic *FTO* variant, the rs9930506 polymorphism, has also been reported to be significantly correlated with *FTO* gene expression. Doaei et al. examined the gene expression pattern of *FTO* in peripheral blood mononuclear cells of obese Iranian male adolescents with different rs9930506 genotypes and found that *FTO* had high expression among the AA genotype carriers but low expression among the AG or GG genotype carriers (*p* = 0.017) [34].

#### 2.1.3. The Interaction between *FTO* Polymorphism and Adjacent Genes

*FTO* has several neighboring genes, including three upstream genes, retinoblastoma-like 2 (*RBL2*), AKT-interacting protein (*AKTIP*), and retinitis pigmentosa GTPase regulator-interacting protein-1-like (*RPGRIP1L*), and three downstream genes, Iroquois homeobox protein 3 (*IRX3*), *IRX5*, and *IRX6*. Most of these adjacent genes have been shown to be correlated with the development of MetS or changes in metabolic parameters associated with MetS [35,36,37,38,39,40]. It has also been proposed that the link between *FTO* polymorphisms and MetS may be associated with the abnormal expressions of these adjacent genes.

### 2.2. The rs9939609 Polymorphism of FTO Is Related to Larger WC in Child and Adolescent Obesity

There was strong evidence of a positive correlation of the A allele of the rs9939609 polymorphism and a greater WC. In a large cohort of Han Chinese children and adolescents aged 7–18 years, Yang et al. observed that WC increased with the TT, TA, and AA genotypes of the rs9939609 polymorphism, in that order (68.2 vs. 70.9 vs. 75.7 cm, *p* = 4.22 × 10^−10^) [41]. This finding was replicated in several other studies assessing the correlation between the rs9939609 polymorphism and WC in children and adolescents, including Europeans [42,43,44,45,46,47,48], East Asians [49,50,51,52], Latin Americans [53,54,55], and North Americans [56]. Notably, Lauria et al. conducted a cross-sectional and longitudinal study to explore the relationship between the rs9939609 polymorphism and obesity-related characteristics in a huge cohort of 16,224 children from eight European nations, and the obtained results displayed that the two-year variation of WC increased with the TT, TA, and AA genotypes, in that order (3.9 vs. 4.3 vs. 4.7 cm, *p* = 0.0007) [45].

Reuter et al. conducted a longitudinal study among Brazilian schoolchildren and followed them for three years to investigate the longitudinal changes in the obesity indexes of the subjects with different rs9939609 genotypes and found that the relative risk of developing abdominal obesity (defined as WC > 75th percentile and standardized by age and sex) was 1.66 times higher among the A allele carriers compared to the TT homozygotes [57]. In a cohort of Chinese children and adolescents aged 6–15 years, Liang et al. observed that the subjects with the AA or AT genotype of the rs9939609 polymorphism had a higher average WC than those with the TT genotype (68.92 vs. 64.25 cm, *p* = 0.036) [58]. The relation between the rs9939609 polymorphism and WC was confirmed in many other cohorts of children and adolescents, including Europeans [59,60,61,62,63,64,65], Asians [66,67], Latin Americans [68,69,70,71], and North Americans [72]. In all these studies, the A allele of the rs9939609 polymorphism was consistently associated with a higher WC in children and/or adolescents. Notably, in a group of Chilean children aged 6–11 years, Molina-Luque and colleagues demonstrated that the prevalence of MetS increased with the TT, TA, and AA genotypes of the rs9939609 polymorphism, in that order (20.2% vs. 25.4% vs. 44.7%, *p* = 0.006) [73].

However, several studies failed to detect a link between the rs9939609 polymorphism and WC in children and adolescents [74,75,76,77]. The inconsistent observations from these studies may be a combination effect of the rs9939609 polymorphism and non-genetic factors, such as sex, sleep duration, screen time, breastfeeding, and ponderal index, on WC. This is supported by data from a cohort study of Portuguese children aged 3–11 years, which displayed that the A allele of the rs9939609 polymorphism was associated with a higher WC in girls (β = 1.91, *p* = 0.005) but not in boys [78]. Moreover, a cross-sectional study in a group of Brazilian children and adolescents that investigated whether physical activity, sleep duration, and screen time affected the association between the rs9939609 variant and WC revealed that the A allele carriers had a higher mean WC than the TT homozygotes in the subgroup with a shorter sleep duration or more screen time everyday [79]. Research on a cohort of Spanish children aged 5–9 years further demonstrated that a decrease in sleep duration was associated with an increase in WC in the children with the TT genotype, but not in those with the AA or AT genotype [80].

Dedoussis and teammates [81] examined the interaction effect of the rs9939609 polymorphism with breastfeeding on WC in two independent cohorts of European children, and obtained a consistent result that the A allele is correlated with a greater WC only in those with less than one month of breastfeeding (*p* < 0.05). Quevedo Alves et al. [82] investigated the interaction effect of the rs9939609 polymorphism with ponderal index at birth on WC in a group of Brazilian children and adolescents aged 6–17 years and found that the subjects with the AA genotype had a higher average WC than those with the AT or TT genotype in the lower tertile (β = 4.40, *p* = 0.048), but not in the middle or upper tertile, of the ponderal index.

#### 2.2.1. Mechanism of rs9939609 Polymorphism Affects Pediatric WC

The mechanisms of action by which *FTO* polymorphisms increase abdominal obesity in childhood and adolescence could be that *FTO* polymorphisms lead to aberrant expression of the *FTO* gene, which in turn disturbs the methylation status of *FTO*-targeted mRNAs, leading to increased food intake and adipogenesis and decreased energy expenditure and thermogenesis, ultimately causing abdominal obesity (Figure 2). Indeed, several studies demonstrated that the minor allele carriers of the *FTO* rs9939609 and rs9930506 polymorphisms had higher expression levels of *FTO* mRNA than non-carriers in blood cells or tissue biopsies [31,32,34]. The target genes of *FTO*, such as SCD which is a key enzyme in adipogenesis and ghrelin which is an important regulator of appetite, are indeed influenced by *FTO* and its polymorphisms. Skuladottir et al. [29] found that the AA genotype carriers of the rs9939609 polymorphism exhibited higher levels of SCD mRNAs than those with the TT genotype in a population of young healthy subjects. *FTO* has also been shown to target the mRNAs of a series of other key genes closely related to obesity, including the adipogenic genes of sterol regulatory element-binding protein 1c (SREBP1c) [83,84,85], carbohydrate responsive element-binding protein (ChREBP) [85], perilipin 5 (PLIN5) [86], and RUNX1 [87], and the lipolytic genes, including peroxisome proliferator-activated receptor alpha (PPARα) [88], peroxisome proliferator-activated receptor gamma (PPARγ) [89], PPARγ coactivator 1α (PGC1α) [90], apolipoprotein E (APOE) [91], hypoxia-inducible factor 1 alpha (HIF1α) [92], and uncoupling protein 1 (UCP1) [93]. *FTO* is highly expressed in the hypothalamus, which has an important role in regulating appetite and satiety [94,95,96]. Ghrelin is a stomach hormone that is released during food restriction and has the properties of stimulating appetite and promoting energy intake. Karra et al. systematically examined *FTO* transcription, ghrelin mRNA methylation status, and ghrelin expression pattern in peripheral blood cells collected from individuals with different rs9939609 genotypes and found that the subjects with the AA genotype had increased *FTO* transcripts, decreased ghrelin mRNA m6A methylation, and increased ghrelin mRNA level as compared to the TT homozygotes [32]. Several studies have demonstrated that *FTO* polymorphisms are highly associated with appetite, satiety, and energy intake in children and adolescents. Wardle et al. offered palatable food to British children aged 4–5 years shortly after having eaten a meal to examine the effect of the rs9939609 polymorphism on appetite and found that food intake increased with the TT, TA, and AA genotypes, in that order (30.00 vs. 37.93 vs. 39.95 g, *p* = 0.032) [97]. In a second cohort study with British children aged 8–11 years, the researchers further found that children with the AA genotype of the rs9939609 polymorphism had reduced satiety responsiveness scores (2.55 vs. 2.67, *p* = 0.008) compared to the TT homozygotes [48]. The link between the A allele of the rs9939609 polymorphism and increased appetite [98,99,100,101,102,103] as well as decreased satiety [100,101,102,103] was further supported by several cohort studies with children and adolescents from various areas of the world. It has been further proposed that the correlation between *FTO* polymorphisms and appetite may be gender dependent. A study on a group of British adolescents aged 14–16 years showed that the A allele of the rs1558902 polymorphism confers a higher risk for binge eating in girls (OR = 1.30, *p* = 3.3 × 10^−4^), but not in boys [104]. Therefore, it may be reasonable to speculate that increased appetite and decreased satiety result in increased total energy intake in children and adolescents.

A meta-analysis of about sixteen thousand children and adolescents aged 1–18 years from fourteen cohorts that examined the association between the rs9939609 polymorphism and total energy intake found that the A allele is correlated with a higher total energy intake (effect per allele = 14.3 kcal/day, *p* = 6.5 × 10^−4^) [105]. Consistently, Cecil et al. demonstrated that the A allele of rs9939609 polymorphism was significantly associated with increased energy intake independently of body weight in a group of Scottish children aged 4–10 years [106]. These results indicate that the A allele carriers consumed more food due to their better appetite. In several other populations of children and adolescents from Europe and Canada [107,108,109], researchers also demonstrated a significant association of the rs9939609 and rs1421085 polymorphisms with total energy intake. Increased energy intake in children and adolescents with specific *FTO* genotypes may be due to their preference for high-energy foods such as those containing fat. Tanofsky-Kraff et al. found that the A allele carriers of the rs9939609 polymorphism had a higher energy intake from fat than did the TT homozygotes in a sample of American children and adolescents aged 6–19 years [102]. This observation was confirmed in a group of British children aged 7 years, which showed that children carrying the A allele of the rs9939609 polymorphism consumed more energy and fat than those not carrying this allele [108].

The *FTO* rs9939609 polymorphism has also been linked with elevated levels of leptin [110], a major appetite-suppressing hormone secreted primarily from adipocytes. Circulating leptin concentrations positively correlate with adipose tissue TG levels, and elevated leptin levels are sensed by the hypothalamus and brainstem as an indicator of sufficient energy reserve, consequently suppressing hunger. Obese individuals typically present reduced leptin sensitivity, causing an ineffective satiety response and excessive hunger. *FTO* works in concert with calmodulin-dependent kinase II (CaMKII) to prolong CREB phosphorylation, consequently modulating the expressions of brain-derived neurotrophic factor (BDNF) and neuropeptide Y receptor Y1 (NPY1R) which are involved in energy homeostasis and lipid metabolic processes [111].

The associations between *FTO* polymorphisms and MetS components may also be mediated by energy expenditure in children and adolescents. Velders et al. examined the effect of the rs9939609 polymorphism on energy expenditure in a population of Dutch preschool children aged 4 years and observed that the A allele is significantly associated with reduced physical activity [103]. This finding was replicated among Indian children with a mean age of 10.3 years, which showed that children with the AA genotype of the rs9939609 polymorphism had the least physical activity and highest frequency in the obesity group [112]. This is in line with the notion that sedentary behavior and cardiorespiratory fitness are associated with the genetic predisposition of children with obesity and MetS. Interestingly, “screen time”, defined as the amount of time spent in front of screen-based devices (e.g., televisions, computers, and videogame devices), was found to be a moderator between *FTO* rs9939609 and the development and progression of childhood and adolescent MetS. Individuals with the rs9939609 AA genotype had low cardiorespiratory fitness, which was relevant to high screen time (designated as 378 min per day or more) [113]. However, researches from Wardle et al. and Cecil et al. showed no significant association between the *FTO* genotype and any markers of physical activity [97,106]. Therefore, further research on the relationship between *FTO* polymorphisms and energy expenditure in children and adolescents may be needed before a conclusive link can be established.

#### 2.2.2. Other *FTO* Polymorphisms Associated with Abnormal WC in Children and Adolescents

Apart from the rs9939609 polymorphism, several other *FTO* intronic polymorphic loci, including rs17817449 (intron 1), rs1421085 (intron 1), rs8050136 (intron 1), rs1558902 (intron 1), rs7206790 (intron 1), rs1861868 (intron 1), and rs11644943 (intron 8), were also examined and shown to be significantly correlated with WC in children and adolescents. In an in vitro study, isogenic allelic series for the rs8050136 and rs1421085 polymorphisms were engineered by using a clustered regularly interspaced short palindromic repeats (CRISPR)/CRISPR-associated protein-9 (Cas9) system in human ESC-derived neurons, and the data suggested that the neurons with the CC genotype of both loci were associated with a 20–30% reduction in *FTO* expression [114]. Additionally, Claussnitzer et al. demonstrated that the rs1421085 polymorphism is located within a conserved motif of AT-rich interaction domain 5B (ARID5B), and the C allele disrupts the ARID5B motif [115]. The C allele led to two times higher expression of *IRX3* and *IRX5* during the early stage of adipocyte differentiation and repair of the ARID5B motif by the CRISPR/Cas9 system in primary adipocytes restored *IRX3* and *IRX5* repression [115]. Furthermore, the rs1421085 SNP was found to be correlated with physical activity among female children and adolescents, with higher physical activity scores in the CC homozygotes than in the TT homozygotes [107].

In silico bioinformatics and chromatin immunoprecipitation analysis identified that the rs8050136 polymorphism is located within the binding site of cut-like homeobox 1 (CUX1), which as a transcription factor can regulate the expression of both *IRX3* and *RPGRIP1L* [116]. This was further validated in an in vitro study, which revealed that the neurons with the CC genotype of the rs8050136 polymorphism had a 20–30% reduction in *RPGRIP1L* expression compared to the AA homozygotes [114]. Doaei et al. examined the gene expression pattern of *IRX3* in a group of obese Iranian male adolescents with different rs9930506 genotypes and found that the AA homozygotes had significantly upregulated transcripts of *IRX3* as compared to the AG or GG genotype carriers (*p* = 0.017) [34]. Jowett et al. found that the rs8050136 polymorphism is strongly correlated with the expression of *RBL2* in human brain samples (*p* = 2.7 × 10^−5^) [117].

The rs17817449 (g.53779455T>G) polymorphism is located approximately 7.2 kb upstream of the rs9939609 polymorphism and formed by a transversion from ancestral allele thymine (T) to guanine (G). Barseem et al. evaluated the association of the rs17817449 polymorphism with obesity indexes among obese Egyptian children and adolescents aged 7–18 years and found that the GG genotype carriers had a higher average WC compared with non-carriers (88.3 vs. 84.1 cm, *p* = 0.046) [118]. This finding was confirmed by Dedoussis and colleagues in a group of Greek children, which displayed that the G allele of the rs17817449 polymorphism is associated with a greater WC (*p* = 0.012) [81]. The rs1421085 (g.53767042T>C) polymorphism is positioned about 20 kb upstream of the rs9939609 polymorphism and is formed by a transition from thymine (T) to cytosine (C). Albuquerque et al. assessed the correlation of the rs1421085 polymorphism with obesity-related traits in a sample of Portuguese children aged 6–12 years and revealed that the WC increased with the TT, TC and CC genotypes, in that order (66.0 vs. 67.5 vs. 68.5 cm, *p* = 0.007) [42]. A similar conclusion was drawn in a group of Mexican children, and the research group demonstrated that the subjects with the CT genotype of the rs1421085 polymorphism had a higher mean WC than those with the TT genotype (71.35 vs. 67.54, *p* < 0.01) [119]. Additionally, the A allele of the rs1861868 polymorphism [42], C allele of the rs8050136 polymorphism [72], A allele of the rs1558902 polymorphism [120], G allele of the rs7206790 polymorphism [121], and T allele of the rs11644943 polymorphism [121] were all shown to be significantly associated with a higher mean WC in children and adolescents.

### 2.3. FTO Polymorphisms Regulate BP in Children and Adolescents

Hypertension, the chronic elevation of blood pressure, is a common feature of metabolic syndrome, arising from the narrowing of blood vessels associated with scar tissue formation or the excessive deposition of low/very-low-density lipoprotein (LDL/VLDL) cholesterol on the inner arterial wall. Hypertension is a major risk factor for cardiovascular disease and increases the risk of stroke. The rs9939609 variant has been frequently reported to be correlated with hypertension in children and adolescents, whilst several other intronic polymorphic loci in *FTO*, such as rs1421085, rs17817449, and rs8050136, have also been reported, although not as often as the rs9939609 polymorphism.

#### 2.3.1. The Link between Hypertension and *FTO* rs9939609 Polymorphism in Children and Adolescents

Molina-Luque et al. carried out a cross-sectional study in a sample of Chilean children aged 6–11 years, and the data suggested that the subjects with the AA or AT genotype of the rs9939609 polymorphism had a greater prevalence of hypertension than those with the TT genotype [73]. García-Solís and colleagues also made a similar observation and found that the AA genotype carriers of the rs9939609 polymorphism had higher levels of SBP (102.5 vs. 98.3 mmHg, *p* < 0.001) and DBP (69.3 vs. 67.1 mmHg, *p* < 0.01) than those with the TT genotype in a group of Mexican children aged 8–13 years [122]. The associations of the A allele of the rs9939609 polymorphism with high BP were replicated and confirmed in multiple pediatric cohort studies involving Polish [63,64,65], Chinese [50,51,123], and Finnish participants [43]. Notably, the correlation between the rs9939609 polymorphism and BP was shown to have an allele-dependent effect. In a large cohort of Han Chinese children and adolescents, Yang et al. found that both SBP (106.7 vs. 108.3 vs. 113.3 mmHg, *p* = 2.12 × 10^−7^) and DBP (65.3 vs. 65.9 vs. 67.7 mmHg, *p* = 0.008) increased with the TT, TA, and AA genotypes of the rs9939609 polymorphism, in that order [41]. These trends were also shown in another group of Chinese children and adolescents aged 6–18 years, in which both SBP (107.1 vs. 108.7 vs. 109.2 mmHg, *p* = 1.41 × 10^−4^) and DBP (67.6 vs. 68.4 vs. 69.3 mmHg, *p* = 0.01) were elevated with the TT, TA, and AA genotypes of the rs9939609 polymorphism, in that order [52].

The correlation between the rs9939609 polymorphism and BP may be modulated by body weight and sleep duration in children and adolescents. Xi et al. examined the relationship of the rs9939609 polymorphism with BP in a population of Chinese children and adolescents aged 6–18 years and observed that the A allele of the rs9939609 polymorphism confers a higher risk for hypertension (OR = 1.35, *p* = 0.001), but this relationship existed only in the obesity group, not in the normal weight group [51]. Short sleep duration has been reported to predispose children and adolescents to hypertension [124,125]. Prats-Puig et al. examined whether this relation is modulated by the rs9939609 polymorphism in a cohort of Spanish children and found that decreasing sleep duration is associated with increasing SBP (β = −1.934, *p* = 0.015) in children with the TT genotype, but not in those with the AA or AT genotype [80].

#### 2.3.2. Other *FTO* Polymorphisms in the Pathogenesis of Hypertension in Children and Adolescents

The rs1558902 (g.53769662T>A) polymorphism is localized about 17 kb upstream of the rs9939609 polymorphism and formed by a transversion from thymine (T) to adenine (A). A study assessed the contribution of the rs1558902 polymorphism to metabolic abnormalities in a Chinese pediatric population and found that the A allele of this locus is associated with a higher mean level of SBP (β = 1.491, *p* = 0.001) [120]. The rs8061518 (g.53827112A>G) polymorphism is a variant situated within intron 3 of *FTO* and formed by a transition from adenine (A) to guanine (G). Olza et al. conducted a multicenter case–control study to systematically investigate the association of 52 polymorphisms in *FTO* with inflammatory and metabolic parameters among Spanish children and adolescents aged 6–15 years. They found that DBP decreased with the AA, AG, and GG genotypes of the rs8061518 polymorphism, in that order (66 vs. 64 vs. 61 mmHg, *p* = 0.006) [47], indicating that G is a protective allele for hypertension in childhood. Chen and colleagues explored the relationships of the rs1421085, rs17817449, rs8050136, and rs3751812 polymorphisms within intron 1 of *FTO* with BP in a group of Chinese children and adolescents and found that the minor allele carriers of these variants had significantly higher levels of SBP than non-carriers [123].

### 2.4. FTO Polymorphisms Regulate Lipid Metabolism in Children and Adolescents

Dyslipidemia, characterized as the imbalance of circulating lipids, is a major hallmark of metabolic syndrome. Several cross-sectional studies demonstrated a significant association between the rs9939609 polymorphism and serum TG as well as HDL-C levels in childhood and adolescence. In addition, the rs1421085, rs17817449, and rs6499640 polymorphisms have also been investigated among children and adolescents and identified to be significantly correlated with serum TG and/or HDL-C levels.

#### 2.4.1. The rs9939609 Polymorphism Increases Risk of Hypertriglyceridemia

Todendi and colleagues examined the influences of the rs9939609 polymorphism on anthropometric and metabolic characteristics in a large cohort of Brazilian children and adolescents and found that the A allele carriers had a higher prevalence of hypertriglyceridemia than the subjects with the TT genotype (28.20% vs. 23.33%, *p* = 0.033) [54]. Similarly, Luczynski et al. observed that the AA homozygotes of the rs9939609 polymorphism had a higher average level of TG than the subjects with the AT or TT genotype in a group of Polish children and adolescents [63]. These findings were confirmed in two other Latin American cohort studies with children aged 4–13 years, which consistently revealed that that the AA homozygotes of the rs9939609 polymorphism had greater levels of TG than the subjects with the AT or TT genotype (*p* < 0.05 for both) [73,126]. They also suggested the possibility of an allele-dependent effect of the rs9939609 polymorphism on serum TG levels. Inandiklioğlu et al. demonstrated that the serum TG levels increased with the TT, TA, and AA genotypes of the rs9939609 polymorphism in a group of obese Turkish children and adolescents aged 3–17 years, in that order (1.21 vs. 1.37 vs. 1.89 mmol/L, *p* = 0.004) [127]. A similar finding was obtained in a study on a group of south Brazilian children and adolescents [53].

Several other polymorphic loci in intron 1 of *FTO*, including the rs1421085, rs17817449, and rs6499640 polymorphisms, have also been shown to be associated with TG among children and adolescents. Cao and teammates assessed the association between the rs1421805 polymorphism and metabolic indexes in a group of Chinese children and adolescents and observed that the C allele carriers had a higher mean level of TG than the TT homozygotes (1.01 vs. 0.91 mmol/L, *p* = 0.05) [128]. Moreover, Inandiklioğlu et al. examined the relation between the rs1421085 polymorphism and serum TG levels in a group of obese Turkish children and adolescents aged 3–17 years and found that TG levels increased with the TT, TC and CC genotypes, in that order (1.21 vs. 1.38 vs. 1.83 mmol/L, *p* = 0.01) [127]. Additionally, two other *FTO* intronic variants, rs17817449 and rs6499640, have also been reported to be associated with higher plasma TG levels. In a cohort of Romanian children and adolescents aged 2–17 years, Duicu et al. found that the G allele carriers of the rs17817449 polymorphism had a higher average level of TG than the subjects with the TT genotype (1.12 vs. 0.39 mmol/L, *p* = 0.003) [129]. Gao et al. investigated the association of the rs6499640 polymorphism with lipid levels in a group of Chinese children and adolescents and observed that the subjects with the GG or GA genotype had a higher average level of TG than those with the AA genotype among girls (0.94 vs. 0.75 mmol/L, *p* = 0.001), but not in boys [130].

#### 2.4.2. *FTO* Polymorphisms Are Involved in HDL Metabolism in Children and Adolescents

An increase in TG is usually accompanied by a decrease in HDL-C in blood. Molina-Luque et al. conducted a cross-sectional study to examine the relation between the rs9939609 polymorphism and MetS components in a group of Chilean children aged 6–11 years and found that the AA homozygote of the rs9939609 polymorphism had lower average level of HDL-C than the subjects with the AT or TT genotype (1.17 vs. 1.34 mmol/L, *p* = 0.001) [73]. A study with a group of Polish children and adolescents further demonstrated that subjects with the AA genotype of the rs9939609 polymorphism had a marginally insignificantly lower average level of HDL-C than those with the AT or TT genotypes (1.22 vs. 1.31 mmol/L, *p* = 0.07) [63]. The rs9939609 polymorphism appeared to have an allele-dependent impact on HDL-C in a cohort study with obese Turkish children and adolescents because the serum HDL-C levels decreased with the TT, TA, and AA genotypes, in that order (1.25 vs. 1.09 vs. 1.07 mmol/L, *p* = 0.003) [127]. The effect of another *FTO* polymorphism, rs1421085, on HDL-C was also observed to be allele dependent, indicated by the higher serum HDL-C in the TT genotype and lower level in the CC genotype in a group of Turkish children and adolescents [127]. In contrast, Wang and colleagues reported that children with the CC genotype of the rs1421085 polymorphism had the highest average level of HDL-C as compared to the subjects with the CT or TT genotype (1.21 vs. 1.09 vs. 1.15 mmol/L, *p* = 0.022) in a group of Chinese children aged 3–6 years [131]. These data indicated that *FTO* polymorphisms are involved in HDL metabolism.

### 2.5. FTO Polymorphisms Modulate Blood Glucose Levels in Children and Adolescents

FBG is a clinical marker used in the diagnosis of type II diabetes, a metabolic syndrome characterized by insulin resistance and/or hyperglycemia and commonly associated with high-fat diets and obesity. Research investigating the association between *FTO* polymorphisms and FBG has mainly been focused on the rs9939609 polymorphism whilst other *FTO* polymorphisms have rarely been reported by the scientific community.

#### 2.5.1. The rs9939609 Polymorphism in Hyperglycemia and Diabetes

Yang et al. assessed the relation between the rs9939609 variant and metabolic parameters in a large sample of Han Chinese children and adolescents and found that subjects with the AA genotype of the rs9939609 polymorphism had a higher average level of FBG than those with the AT or TT genotype (4.9 vs. 4.7 mmol/L, *p* = 0.012) [41]. In another cohort of Chinese children with a mean age of 9.79 years, the researchers reported a similar observation which showed that the A allele carriers of the rs9939609 polymorphism had a higher average level of FBG than the subjects with the TT genotype (4.88 vs. 4.68 mmol/L, *p* = 0.026) [132]. In a cohort of Brazilian children and adolescents aged 8–17 years, Nascimento and colleagues demonstrated that the AA genotype of the rs9939609 variant is correlated with a higher level of FBG (*p* = 0.003) [133]. Interestingly, gender may play a role in the link between the rs9939609 polymorphism and FBG. In a group of Swedish adolescents with a mean age of 15 years, Jacobsson et al. observed that the AA homozygotes of the rs9939609 polymorphism had a higher average level of glucose than subjects with the AT or TT genotype in girls (4.9 vs. 4.7 mmol/L, *p* = 0.0248), but not in boys [134]. In contrast, newborns with mothers carrying the AA or AT genotype had a lower average level of FBG than those with mothers carrying the TT genotype (3.63 vs. 4.68 mmol/L, *p* = 0.015) [135]. Moreover, rs9939609 has been implicated in the development of gestational diabetes mellitus (GDM). However, research outcomes from these studies are inconsistent [136,137,138]. The reason for the inconsistent observations among different studies may be due to the relatively low frequency of the A allele of rs9939609 among some populations, indicating the complexity of the role of the rs9939609 polymorphism in blood glucose regulation.

#### 2.5.2. Other *FTO* Polymorphisms in Hyperglycemia

The rs9930506 (g.53796553A>G) polymorphism is localized within intron 1 of *FTO* approximately 10 kb downstream of the rs9939609 polymorphism and formed by a transition from ancestral allele adenine (A) to guanine (G). Li and teammates examined the effects of the rs9930506 polymorphism on metabolic traits in a cohort of Chinese children with an average age of 9.68 years and revealed that the G allele carriers had a greater average level of FBG than the children with the AA genotype (4.93 and 4.69 mmol/L, *p* = 0.017) [139]. The rs17817449 polymorphism is also positioned in intron 1 of *FTO*, which is associated with increased fasting glucose in obese Egyptian children and adolescents aged 7–18 years. The FBG was increased in a pattern associated with TT, TG, and GG genotypes (5.97, 6.02, and 6.34 mmol/L, respectively, *p* = 0.029) [118].

## 3. Advances in Prevention and Treatment of MetS in Children and Adolescents

Lifestyle during early development, childhood, and adolescence has a marked and profound effect on future health and fitness [140,141]. Ample evidence has demonstrated that environmental and lifestyle factors during early life, including diet, dyslipidemia, hypertension, MetS, type II diabetes, and obesity, are associated with cardiovascular health in adulthood. A childhood dietary intervention study demonstrated that a high-fiber, low-fat diet provided to hypercholesterolemic children induced lasting benefits evident in adulthood [142], which may suggest that cardiovascular diseases could be rooted in childhood/adolescence. Therefore, it is imperative that lifestyle intervention should start at an early stage. Children carrying unfavorable genetic polymorphisms of the *FTO* gene are particularly at risk of adulthood cardiovascular disease and consequently should receive intervention at the earliest possible stage.

Currently, the medical treatments for MetS are very limited, and lifestyle interventions are usually recommended to improve and prevent MetS in children and adolescents [143,144,145]. Lifestyle interventions consist of a reduction in energy intake and an elevation in physical activity, as well as the use of behavioral therapy [146]. The lifestyle intervention program proposed by Foster and teammates has been widely recognized and applied to improve MetS components in children and adolescents [147]. Foster’s lifestyle intervention program includes several stages such as Prevention Plus, structured weight management, and comprehensive multidisciplinary intervention. Prevention Plus addresses the importance of a healthy diet and daily physical activity. Structured weight management involves a structured physical activity program, a structured approach to diet, and a monthly monitoring schedule. Comprehensive multidisciplinary intervention focuses on structured behavioral intervention with behavioral counselors, dieticians, and activity specialists and a weekly monitoring schedule. Most of the lifestyle interventions in children and adolescents lasted for 6 to 24 months and led to significant improvements in MetS components [143,144,145]. To better treat childhood MetS, a multidisciplinary team should also include pediatricians and psychologists.

Pharmacotherapy and bariatric surgery must be considered if lifestyle interventions are not able to solve the problems of severe MetS and its complications in children and adolescents. Although several new drugs for weight loss have been put on the market in the past few decades, most of them were quickly withdrawn due to ineffectiveness or severe side effects, and none of them was suitable for children and adolescents [148]. Metformin was approved for use in children by the US Food and Drug Administration, but it is only permitted to be used in children with type II diabetes mellitus and not in those with insulin resistance or impaired glucose tolerance. Some incretion hormones, such as glucagon-like peptide 1, are currently under development or in clinical trials to treat childhood obesity, but to date none of these drugs has been approved for marketing anywhere in the world. For those children and adolescents with morbid obesity and severe metabolic disorders, bariatric surgery can be used when all other conservative treatment approaches fail [149]. However, bariatric surgery requires careful use in children and adolescents because there are some potential risks involved, such as dumping syndrome, deficiency of lipid-soluble vitamins, electrolyte disturbance, and difficulties in postoperative care. Moreover, there is also a lack of evaluation of long-term efficacy and safety after bariatric surgery in the pediatric population [150].

## 4. Conclusions and Future Perspectives

The increasing incidence of childhood and adolescent obesity and metabolic disease in recent decades represents a concerning increasing clinical burden. Obesity and MetS are multifactorial conditions arising from a complex interaction between genetic and environmental factors. The environmental factors causing MetS are well documented and consist of a combination of poor diet, e.g., high-fat and high-fructose diets, sedentary lifestyle, and exposure to environmental pollutants. However, our knowledge of genetic risk-factors for metabolic syndrome is limited. The research discussed throughout this review provides insight into the impact of genetic polymorphism on the susceptibility of an individual to MetS when the affected gene is a key regulator of human satiety and energy expenditure. Of note, of MetS-associated polymorphisms of *FTO*, rs9939609 is the most extensively studied, whilst several other *FTO* polymorphisms may or may not contribute to childhood and adolescent MetS. There are also contradictory data on the role and extent to which *FTO* polymorphisms increase susceptibility to MetS. Some authors have attributed these discrepancies to the rarity of some polymorphic alleles and differences in the frequency of some alleles between different geographic or ethnic populations. More intriguingly, all the major MetS-associated polymorphisms in *FTO* are found in introns; whether this is a consequence of polymorphisms occurring in exons or promoter regions that results in non-functional proteins or impaired genetic expression is currently unknown. A potential mechanism could be that these polymorphisms interfere with the process of RNA splicing, leading to the upregulation of less favorable *FTO* isoforms. Another possibility is that these polymorphisms regulate unidentified non-coding RNAs (e.g., miRNAs, circRNAs, or lncRNAs) encoded by the introns of *FTO*. Therefore, further research that can provide a direct answer on how these polymorphisms affect the tendency to develop MetS would be meaningful. In addition, advanced genome-editing technology, such as the CRISPR/Cas9 system and base editing technique, could be further applied to correct these harmful polymorphic sites and evaluate their clinical significances in further research.

## Figures and Tables

**Table 1:** Definitions of metabolic syndrome in children and adolescents.

MetS Components	IDF Criteria			NCEP ATP III Criteria
	6–9 Years	10–15 Years	≥16 Years	ND
Abdominal obesity	WC ≥ 90th percentile for age [15]	WC ≥ 90th percentile [16]	WC ≥ 94 cm in boys and ≥ 80 cm in girls [15] or WC ≥ 90 cm in boys and ≥ 80 cm in girls [17]	WC ≥ 102 cm in boys and ≥ 88 cm in girls [16] or WC ≥ 75th percentile for age and sex [17]
Hypertension	ND	SBP ≥ 130 or DBP ≥ 85 mmHg [17]	SBP ≥ 130 or DBP ≥ 85 mmHg [17]	SBP ≥ 130 mmHg [16] or SBP ≥ 90th percentile for age and sex [17]
Hypertriglyceridemia	ND	TG ≥ 1.7 mmol/L [16]	TG ≥ 1.7 mmol/L [17]	TG ≥ 1.7 mmol/L [17]
Hypo-high-density lipoproteinemia	ND	HDL-C ≤ 1.03 mmol/L [16]	HDL-C ≤ 1.03 mmol/L in boys and ≤ 1.29 mmol/L in girls [17]	HDL-C ≤ 1.03 mmol/L [16]
Hyperglycemia	ND	FBG ≥ 5.6 mmol/L [16]	FBG ≥ 5.6 mmol/L [17]	FBG ≥ 6.1 mmol/L [16]
Diagnostic criteria	Abdominal obesity and any two of the other components must be present			At least three components are present

IDF, the International Diabetes Federation; NCEP ATP III, the National Cholesterol Education Program’s Adult Treatment Panel III; WC, waist circumference; SBP, systolic blood pressure; DBP, diastolic blood pressure; TG; triglyceride; HDL-C, high-density lipoprotein cholesterol; FBG, fasting blood glucose; ND, not declared.

## Data Availability

Data sharing not applicable.
